# Knowledge and practice of preconception care among women in southeastern Mexico

**DOI:** 10.15649/cuidarte.3512

**Published:** 2024-08-02

**Authors:** Nora Hernández-Martínez, Andrea Paola Pérez-Chablé, Velia Margarita Cárdenas-Villarreal, Norma Edith Cruz-Chávez, Jesús Melchor Santos-Flores

**Affiliations:** 1 Universidad Autónoma de Nuevo León, México. E-mail: nora.hernandezmz@uanl.edu.mx Universidad Autónoma de Nuevo León Universidad Autónoma de Nuevo León Mexico nora.hernandezmz@uanl.edu.mx; 2 Universidad Autónoma del Campeche. E-mail: andreapprzc@gmail.com Universidad Autónoma de Campeche Universidad Autónoma del Campeche Mexico andreapprzc@gmail.com; 3 Universidad Autónoma de Nuevo León, México. E-mail: velia.cardenasvl@uanl.edu.mx Universidad Autónoma de Nuevo León Universidad Autónoma de Nuevo León Mexico velia.cardenasvl@uanl.edu.mx; 4 Universidad Autónoma de Nuevo León, México. E-mail: norma.cruzch@uanl.edu.mx Universidad Autónoma de Nuevo León Universidad Autónoma de Nuevo León Mexico norma.cruzch@uanl.edu.mx; 5 Universidad Autónoma de Nuevo León, México. E-mail: jsantosen0347@uanl.edu.mx Universidad Autónoma de Nuevo León Universidad Autónoma de Nuevo León Mexico jsantosen0347@uanl.edu.mx

**Keywords:** Preconception Care, Women, Healthy Lifestyles, Reproductive Health, Knowledge, Atención Preconceptiva, Mujeres, Estilos de Vida Saludable, Salud Reproductiva, Conocimiento, Cuidados Pré-Concepcional, Mulheres, Estilo de Vida Saudável, Saúde Reproductiva, Conhecimento

## Abstract

**Introduction:**

Preconception care is intended to increase the chances of a favorable perinatal outcome; little is known about it in low- and middle income countries in Latin America.

**Objective:**

To evaluate the knowledge and practices of PCC and its relationship with sociodemographic and obstetric characteristics in women of childbearing age in southeastern Mexico.

**Materials and Methods:**

A cross-sectional study was conducted with 138 women attending health care centers. Consecutive sampling was used, and data were collected with a structured questionnaire. Data analysis involved the calculation of frequencies, percentages, and inferential tests.

**Results:**

Women's APC knowledge score obtained a mean of 13.3 ± 3.24 and for practices a mean of 8.13 ± 3.44 scores considered low for both. Knowledge of APC obtained a relationship with schooling (rs.460, p < .05), monthly economic income (rs =.334, p < .05). In addition, a positive and significant relationship was obtained between knowledge and practice of CPA (rs=.267, p < .05).

**Discussion:**

These findings are consistent with results reported in studies conducted in low- and middle-income countries.

**Conclusions:**

There is a relatively low level of knowledge and adoption of CPA practices in women of childbearing age in southern Mexico, and it is related to education and socioeconomic income, which places them in a population group at high risk for maternal and child health problems.

## Resumen

**Introducción:**

La atención preconcepcional tiene la finalidad de aumentar las posibilidades de un resultado perinatal favorable, se conoce poco de ella en países de bajos y medianos ingresos en América Latina.

**Objetivo:**

Evaluar el conocimiento y prácticas de la APC y su relación con características sociodemográficas y obstétricas en mujeres en edad fértil en el sureste de México.

**Materiales y Métodos:**

Se realizó un estudio transversal con 138 mujeres que asistieron a centros de atención. Se utilizó un muestreo consecutivo y los datos se recogieron con un cuestionario estructurado. El análisis de datos implicó el cálculo de frecuencias, porcentajes y pruebas inferenciales.

**Resultados:**

La puntuación de las mujeres sobre conocimiento de APC obtuvo una media de 13,3 ± 3,24 y para prácticas una media de 8,13 ± 3,44 puntuaciones consideradas como bajas para ambas. El conocimiento de APC obtuvo una relación con la escolaridad (rs.460, p < ,05), el ingreso económico mensual (rs =,334, p < ,05). Además, se obtuvo una relación positiva y significativa entre conocimiento y práctica de la APC (rs=,267, p < ,05).

**Discusión:**

Estos hallazgos coinciden con los resultados reportados en estudios realizados en países de bajos y medianos ingresos.

**Conclusiones:**

Hay un nivel relativamente bajo de conocimiento y adopción de prácticas de APC en mujeres en edad fértil en el sur de México, y se relaciona con la educación y el ingreso socioeconómico, lo que las coloca en un grupo poblacional de alto riesgo de problemas de salud materno- infantil.

## Resumo

**Introdujo:**

A assistencia pré-concepcional tem como objetivo aumentar as chances de um resultado perinatal favorável; pouco se sabe sobre ela em países de baixa e média renda da América Latina.

**Objetivo:**

Avaliar o conhecimento e as práticas de APC e sua relagáo com características sociodemográficas e obstetrícia em mulheres em idade fértil no sudeste do México.

**Materiais e Métodos:**

Foi realizado um estudo transversal com 138 mulheres frequentadoras de centros de atendimento. Foi utilizada amostragem consecutiva e os dados foram coletados por meio de questionário estruturado. A análise dos dados envolveu cálculo de frequencias, porcentagens e testes inferenciais.

**Resultados:**

A pontuagáo das mulheres no conhecimento da APC obteve média de 13,3 ± 3,24 e para práticas médias de 8,13 ± 3,44, pontuagóes consideradas baixas para ambos. APC obteve relagáo com a escolaridade (rs.460, p < 0,05), renda económica mensal (rs = 0,334, p < 0,05). Além disso, obteve-se uma relagáo positiva e significativa entre o conhecimento e a prática de APC (rs = 0,267, p < 0,05).

**Discussao:**

Os resultados coincidem com os resultados relatados em estudos realizados em países de baixa e média renda.

**Conclusóes:**

Há um nivel relativamente baixo de conhecimento e adogáo de práticas de APC em mulheres no sul do México e está relacionado a educagáo e a renda socioeconómica, o que as coloca num grupo populacional de alto risco para problemas de saúde materno-infantil.

## Introduction

Preparation for a healthy pregnancy, through preconception care and prevention of unplanned pregnancies, has recently gained relevance in research and public health policies as priority health actions^1^. Preconception care (PCC) is defined as a set of actions that aim to promote health in women of reproductive age before pregnancy, to identify and modify biological, behavioral and social risk factors in the woman and her future child. Its ultimate goal is to improve maternal and child health outcomes and well-being, both in the short and long term^2^.

The main risk factors that affect PCC include biological ones such as: parental age, female malnutrition, maternal obesity, multiparity, genetic conditions, chronic and communicable diseases. Psychological factors such as: stress and anxiety, along with the family or relationship environment, are relevant, lack of knowledge about PCC. Social and environmental risks include the consumption of nutrients such as folic acid, drugs, medications, exposure to chemicals, access to health services and poor lifestyles^3^^, ^^4^. It is estimated that 90% of women of reproductive age have at least one modifiable risk factor^1^.

One of the above factors, which has drawn pressing attention to improve preconception health, is obesity, which affects a significant percentage of women of childbearing age and is strongly related to almost all adverse outcomes of pregnancy and childbirth, particularly preeclampsia, gestational diabetes and stillbirth, and has long-lasting consequences for the health of offspring5. Unfortunately, attempts to address the problem through dietary and physical activity interventions since pregnancy have had a negligible effect on immediate and subsequent outcomes^5^^, ^^6^. Together, these findings call for a new approach to improving *preconception health*.

The evidence suggests that the use of PCC before planning a pregnancy (ideally six months before) helps to reduce worldwide the number of women who die from causes related to pregnancy and childbirth, contribute to the reduction of disabilities related to birth defects birth^4^^, ^^7^. Women of childbearing age who wish to become pregnant should be advised on healthy living (weight as close to ideal as possible, exercise, sleep and control of any pre-existing conditions before conceiving) and on how to avoid risk factors for the product. (stress, alcohol, smoking, including secondary smoking, exposure to chemicals and drug use) and should also be advised on the consumption of folic acid supplements with the aim of preventing neural tube defects and the application of vaccines^1^^, ^^2^.

Knowledge of PCC is strongly related to its practice. However, the practice of PCC in women of reproductive age is reported at around 50% in high-income countries^(8)^, and even less in low- and middle-income countries^9^. In contrast, prenatal consultation, although important, is often considered late^10^. Therefore, preventive intervention is invaluable, they are more likely to follow PCC guidelines, and such as socioeconomic level, education and access to health services^11^^, ^^12^. Knowledge can come from health professionals, personal experiences, family or the media^12^. Studies in Europe and Africa have noted that women of childbearing age report low PCC knowledge, while women in high-income countries such as the United States and Canada and China have higher knowledge^13^^, ^^15^. Furthermore, individual women's factors, such as education, use offamily planning, and obstetric history, influence knowledge of PCC^3^^, ^^12^^, ^^14^.

Developed countries have PCC care guides and protocols that help women follow their recommendations^1^. Mexico considered a middle-income country, in the last decade has incorporated actions directly aimed at PCC within the health system to improve care for women of childbearing age and reduce the adverse outcomes of pregnancy and childbirth^15^. However, evidence of the knowledge and practice of women of childbearing age regarding preconception health and associated factors is not known in this population to date. Greater understanding of women's knowledge and practice of preconception health, especially in resource-limited health settings, is needed as it could help formulate interventions to improve overall outcomes for women, mothers and babies. This study aims to evaluate the knowledge and practice of PCC and its relationship with sociodemographic and obstetric characteristics in women of childbearing age in southern of Mexico.

## Materials and Methods

### Type of study

The type of study was a cross-sectional descriptive study with a correlational scope carried out in the first half of 2022^16^.

### Population

The population was represented by women of childbearing age between 18 and 49 years old, beneficiaries who attended the general consultation at first-level public health care centers in the City of Campeche, Mexico.

### Sample and sampling

A sample size of 138 women was estimated, calculated with the G* POWER statistical package version 3.3, considering a significance level of 0.05 estimated for a correlation analysis, with a coefficient of determination of 0.3 with a size medium effect low^17^ and a power of 90%. A consecutive sampling technique was used, in which pregnant women who attended first-level care centers located in the State of Campeche were recruited, until the desired sample size was achieved. The inclusion criteria were women who spoke Spanish and who intended to become pregnant in the next 6 months. Women with hearing impairments and critical illnesses were not eligible for the study.

### Procedure and instruments for collecting information

Participants were recruited by nursing staff previously trained on the objectives of the study and data collection techniques. Once the participant was identified and met the inclusion criteria, she was informed of the objective of the study, that participation was voluntary, and that the information would be confidential. If you agreed to participate, written informed consent was provided for your signature. The questionnaires were applied through interviews.

To evaluate the variables of interest, the Knowledge and Practice of Preconception Care (CPAP) Questionnaire designed for Ethiopian women and available in English was used. This questionnaire was developed based on existing literature on knowledge and recommended practices in women of childbearing age, demonstrating its satisfactory internal consistency (Cronbach's alpha 0.93)^18^. The questionnaire is made up of 32 questions, distributed in four sessions; The first includes sociodemographic data of the woman (5 questions); age in years, marital status (with a partner and without a partner), education (years), occupation (work yes, no) and monthly income (Mexican pesos). The second session (eight questions); I include obstetric data: number of pregnancies, number of living children, whether you have used prenatal care and how many consultations I carried out, whether you have used the hospital service for childbirth care, whether you have used other care services for childbirth, used well-childcare and use of family planning consultation.

The session three evaluates knowledge ofthe PCC (20 questions), related to individual, behavioral and environmental risk factors. The response pattern is dichotomous of “yes” and “no” for all questions, the correct answer was assigned a value of 1 and the incorrect one a value of 0. The score for obtaining ranged from 0-20, the higher the score, the higher. knowledge of PCC. It was also classified by level, from zero to 11 points low, from 12 -14 moderate and 15 to 20 high knowledge of PCC.

Session four addresses the practice of PCC (17 questions), actions that the woman took before planning a pregnancy were evaluated. The response pattern is dichotomous from 1 if it was done and 0 if it was not done. The sum of the answers has a range of 0-17 points, the higher the score, the greater the PCC practice. The total score was classified by level: 0-7 was classified as low, 8-9 was moderate, and 11-17 was classified as high PCC practice.

To adapt the questionnaire to the context of the present study, a translation from English to Spanish was performed, followed by another reverse translation to ensure consistency. Subsequently, a pilot test was carried out to evaluate its consistency. The internal consistency of the CPAP for this study was determined using the Kuder -Richardson coefficient, obtaining satisfactory values of 0.72 and 0.82 for knowledge and practice, respectively^16^.

### Statistical analysis

For this research, the information was processed through the Statistical Package for the Social Sciences (SPSS), version 21 for Windows, frequencies and percentages were calculated, descriptive statistics were used using mean, median and standard deviation. The distribution of normality was also determined through the Kolmogorov test. Smirnov, where no normality of the data was found, so the Spearman correlation coefficient was used. The database was later stored in Mendeley Data^19^.

### Ethical Considerations

The study was approved by the Research and Research Ethics Committee of the Faculty of Nursing of the Autonomous University of Nuevo León (registration number FAEN-M-1905) and adhered to the Regulations of the General Health Law on Research for Health Reform Published DOF 04-02-2014, the participants gave their authorization to participate in the study through written informed consent^20^.

## Results

A total of 138 women of childbearing age were included. The average age of the participants was 25.51 ± 5.66 and an average educational level of 10.70 years ± 3.22. The majority did not work (79.00%) and had an economic income in Mexican pesos of 4724.63 ± 2375.35. Only 36.23% (50) reported having heard about PCC and those who reported having information, 30.43% (42) indicated that it was health personnel who learned it provided (Table 1).

**Table 1:** Descriptive statistics of the individual characteristics of women from southeastern of Mexico.

Characteristic	M ± SD % (n) 138
Civil status	
With partner	79.73 (110)
Single	20.27 (28)
Occupation	
With employment	21.00 (29)
Unemployed	79.00 (109)
Number of from pregnancies	2.05 ± 1.28
Number of children	1.70 ± 1.04
Utilized prenatal care	88.40 (122)
Number of prenatal consultations	6.21 ± 3.04
Uses family planning methods	40.66 (56)
Have you heard about Preconception care?	36.23 (50)
From whom the information was received:	
Family	2.24 (3)
Health institutions	30.43 (42)
Media	0.71 (1)
School	2.24 (3)
Others	0.70 (1)

*n = number of cases (138); % = percentage; M = mean; SD = standard deviation*

Regarding the knowledge of PCC in Table 2, it is observed that the women reported not knowing that untreated health problems such as epilepsy, stress and depression (46.49%) and genetic problems (63.00%) could affect the development of the fetus. Just as a high percentage was unaware of the use of a family planning method before pregnancy (52.20%), screening tests for heredity-related diseases (49.30%) were necessary to prevent pregnancy and product problems.

**Table 2:** Knowledge of PCC of women in southeastern of Mexico

Variable	Yes %(n)	No %(n)
Untreated health problems affect the fetus such as:		
Mellitus diabetes	86.23(119)	13.87(19)
Epilepsy	53.51(74)	46.49(64)
Obesity	73.28(101)	26.72(37)
STIs and HIV	89.12(123)	10.88(15)
Heart diseases including high blood pressure	78.31(108)	21.69(30)
Stress and depression	53.51(74)	46.49(64)
Genetic problem	37.00(51)	63.00(87)
Social and cultural behaviors of parents affect the outcome of pregnancy such as:		
Smoke a cigarette	86.35(133)	13.65(5)
Alcohol consumption	77.13(134)	22.87(4)
Illegal drug use	44.86(131)	55.14(7)
Exposure to environmental risks	79.72(110)	20,28(28)
A woman must:		
Use a family planning method during the period before pregnancy.	47.87(66)	52.13(72)
Get vaccinated against tetanus and rubella before you become pregnant.	69.59(96)	30.41(42)
Undergo testing for medical conditions to assess your health status (weight, blood pressure, anemia, diabetes, and HIV)	75.32(104)	24.68(34)
Avoid consuming drugs (alcohol, smoking cigarettes and marijuana, cocaine or others) before pregnancy,	33.57(129)	66.43(9)
Control weight and consume folic acid before pregnancy.	65.82(91)	34.18(47)
Undergo screening tests for family diseases prior to pregnancy.	50.73(70)	49.27(68)
Create healthy environments (free of radiation, chemicals and stress) before pregnancy.	69.51(96)	30.49(42)

*n = number of cases (138); % = percentage.*

In relation to the practice of PCC, only 38.39% (53) of the women reported attending health institutions before pregnancy. Of these, the most performed action (23.25%) was the application of recommended vaccines. 75.34% (104) of women did not maintain a healthy weight before pregnancy. and 79.73% (110) considered the practice of healthy environments before pregnancy as shown in Table 3.

**Table 3:** Reported PCC practices of women in southeastern of Mexico

Component	Yes %(n)	No %(n)
Visited health institutions before pregnancy	38,39(53)	61.61(85)
Why they visited the institute:		
Take folic acid	17.31(24)	82.69(114)
Being examined and treated for an illness	18.86(26)	81.14(112)
Get a vaccine	23.25(32)	76.75(106)
Receive medical help	22.58(31)	77.42(107)
Use family planning	10.89(15)	89.11(123)
Maintained or adjusted your weight before pregnancy	24.67(34)	75.34(104)
Modified diet	21.95(29)	78.05(109)
Exercised	10,17(14)	89.83(124)
Avoided taking substances (alcohol, tobacco or drugs) before pregnancy	88.37(122)	11.63(16)
Smoke a cigarette	87.77(121)	12.24(17)
Alcohol consumption	82.52(114)	17.48(24)
Illegal drug use	89.13(123)	10.87(15)
Created healthy environments before pregnancy	79.73(110)	20,27(28)
Free of environmental radiation	73.29(101)	26.71(37)
Free of environmental chemicals	75.35(104)	24.65(34)
Free of stressors	50.87(69)	49.13(69)

*n = number of cases (138); % = percentage.*

The total PCC knowledge score obtained an average of 13.33 points (SD = 3.24; range 5 - 19) and for PCC practices an average of 8.13 points (SD = 3.44; range 0 -16). When classifying their scores, it was found that (24.62%) of the participants had a low level of knowledge and (35.51%) reported low practice, as shown in Table 4.

**Table 4:** Level of knowledge and practice of PCC of women in southeastern of Mexico

Variable	%(n) 138
Knowledge	
Low	24.62(34)
Moderate	37.03(51)
High	38.35(53)
Practice	
Low	35.51(49)
Moderate	33.25(46)
High	31.24(43)

*n = number of cases (138); % = percentage.*

In **Table 5**, a relationship between knowledge and PCC practices was reported ( rs =.267, p =0.002) and PCC knowledge with schooling, this means that the higher the schooling, the greater the knowledge and practice of PCC ( rs = .460, p =0.001), economic income ( rs =.334, p =0.001), the higher the economic income, the greater the knowledge and practice of PCC, the number of pregnancies and children ( rs = -.229, p =0.007), due to the number of pregnancies and experience, greater knowledge and practice of PCC for marital status and occupation, no difference was found.

Table 5. Spearman Correlation Coefficient for individual characteristics, knowledge and practice of PCC in study participants

n = number of cases (138); rs = Spearman correlation coefficient; p =observed significance; ** p = < .01; * p = <.05; PCC = Preconception care

## Discussion

In this study, it was observed that women of childbearing age have poor knowledge and practices regarding preconception care (PCC). These findings coincide with results reported in studies conducted in low- and middle-income countries^9^^, ^^11^^, ^^14^, suggesting that governmental and non governmental health organizations should pay attention to raising awareness and implementing measures to improve care. prior to conception^2^.

Although more than half of the women interviewed in this study recognized that PCC is care intended for women of childbearing age, none could respond satisfactorily about it. The level of PCC knowledge and its characteristics and components turned out to be relatively low. It was striking that more than half of the participants were unaware of the importance of prior evaluation of medical conditions such as weight, blood pressure, anemia, diabetes, HIV and genetic conditions, to prevent problems in the fetus and complications during pregnancy and childbirth. These gaps in knowledge of specific preconception medical care topics are in line with previous studies^11^^, ^^13^^, ^^14^^, ^^19^^, ^^21^.

Likewise, a positive and significant relationship was observed between PCC knowledge and the level of education and economic income of the participants. Similar results have been reported by countries such as Ethiopia^13^^, ^^21^ China^15^ and Iran^22^. It is considered that women with a higher level of education can discuss sensitive topics openly and freely and seek information about their health.

The economic factor also has a direct implication on health, since it increases the possibility of having greater access to health services in most cases^11^^, ^^23^^, ^^25^. The results of this study show the need to continue efforts to raise awareness among the population with emphasis on the specific aspects of health issues, the timing and importance of PCC, among others.

Regarding PCC practices, it was observed that 88% of the participants had attended prior prenatal care. Of these, only 38% had visited health institutions before becoming pregnant, however, they were unaware that they should be evaluated in relation to previous illnesses, family planning and start taking folic acid. These percentages are higher than those recorded in previous studies^21^^, ^^26^.

A possible explanation lies in the positive and significant relationship between PCC practice and knowledge found in this study. This is because knowledge of PCC can increase understanding, awareness, and practice of PCC components^11^^, ^^27^. Also, it may be due to poverty, poor attitude towards health among the population and lack of access to health services^19^^, ^^21^. Furthermore, the health personnel responsible for these programs must have the best evidence-based practices to provide the best PCC health care^28^^, ^^30^ aimed mainly at populations with a higher risk of maternal and child health^30^^, ^^32^.

The strength ofthe study lies in being one ofthe first studies to describe the knowledge and practices of PCC in a group of women from southern of Mexico. However, its limitation is that it is a cross sectional design study with a small sample size. It is recommended that future research address the factors that affect the PCC and assess the psychometric properties of the PCC knowledge and practices instrument for reproducibility and validity in various economic and social environments, to have a broader vision of the study phenomenon.

## Conclusion

There is a relatively low level of knowledge and adoption of PCC practices in women of childbearing age in southern of Mexico, and it is related to education and socioeconomic income, which places them in a population group at high risk for maternal and child health problems.

Health personnel at the first level of care play a key role in promoting maternal and child health and, as such, must intensify their efforts to encourage women, especially at the community and primary care levels, to receive this care. and consistently emphasize the importance and benefits of PCC during routine health care visits. PCC practices should be provided through evidence-based protocols and guidelines to improve the health of women and children in low- and middle-income countries where many maternal and child deaths continue to occur and to reduce disparities. and increase equity in global health and development.
